# Infection-related hemolysis and susceptibility to Gram-negative bacterial co-infection

**DOI:** 10.3389/fmicb.2015.00666

**Published:** 2015-06-30

**Authors:** Katharine Orf, Aubrey J. Cunnington

**Affiliations:** Section of Paediatrics, Imperial College LondonLondon, UK

**Keywords:** co-infection, hemolysis, malaria, Bartonellosis, Babesiosis, *Salmonella*, heme oxygenase

## Abstract

Increased susceptibility to co-infection with enteric Gram-negative bacteria, particularly non-typhoidal *Salmonella*, is reported in malaria and Oroya fever (*Bartonella bacilliformis* infection), and can lead to increased mortality. Accumulating epidemiological evidence indicates a causal association with risk of bacterial co-infection, rather than just co-incidence of common risk factors. Both malaria and Oroya fever are characterized by hemolysis, and observations in humans and animal models suggest that hemolysis causes the susceptibility to bacterial co-infection. Evidence from animal models implicates hemolysis in the impairment of a variety of host defense mechanisms, including macrophage dysfunction, neutrophil dysfunction, and impairment of adaptive immune responses. One mechanism supported by evidence from animal models and human data, is the induction of heme oxygenase-1 in bone marrow, which impairs the ability of developing neutrophils to mount a competent oxidative burst. As a result, dysfunctional neutrophils become a new niche for replication of intracellular bacteria. Here we critically appraise and summarize the key evidence for mechanisms which may contribute to these very specific combinations of co-infections, and propose interventions to ameliorate this risk.

## Introduction

An association between infection-related hemolysis and bacterial co-infection has been known for almost a century (Giglioli, 1929), but accumulating evidence now shows a clear causal link. Most of this evidence comes from malaria and non-typhoidal *Salmonella* (NTS) co-infections (Takem et al., 2014), but Oroya fever (*Bartonella bacilliformis* infection) is another hemolytic infection which is strongly associated with Gram-negative bacterial co-infection (Minnick et al., 2014). In this review, we outline the causes and consequences of hemolysis, critically appraise the evidence for an association between infection-related hemolysis and susceptibility to co-infection, and provide an overview of possible mechanistic explanations.

## Hemolysis

**Figure 1:**
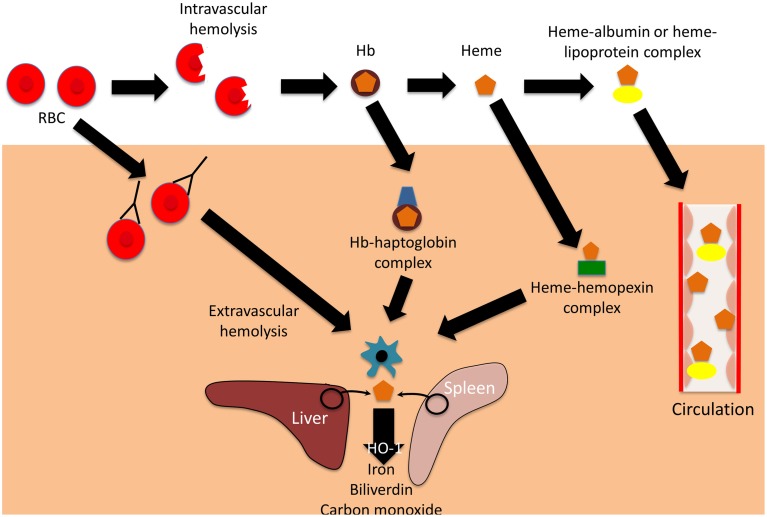
**Mechanisms and consequences of hemolysis**. The fate of the contents of red blood cells (RBCs) depends on whether hemolysis is extravascular or intravascular. Following intravascular hemolysis, hemoglobion (Hb) is bound by haptoglobin and taken up by monocytes and macrophages. When haptoglobin is depleted, heme is released from Hb and is bound by hemopexin. The heme-hemopexin complex is primarily cleared by macrophages and hepatocytes. If hemolysis overwhelms the capacity of both haptoglobin and hemopexin, heme remains within the circulation, weakly binding to albumin and lipoproteins, and can interact with other cell types. In extravascular hemolysis, red blood cells are removed by phagocytic cells, primarily in the spleen and liver. Heme released from both intra- and extravascular hemolysis induces the expression of heme oxygenase-1 (HO-1), which degrades heme to iron, biliverdin, and carbon monoxide.

Hemolysis is the premature destruction of red blood cells (RBCs) before the end of their normal life span, and hemolytic anemia occurs when the production of new RBCs from bone marrow fails to compensate for this loss of RBCs (Guillaud et al., 2012). The causes of hemolysis can be broadly divided into disorders intrinsic or extrinsic to the RBC, and the location of hemolysis can be subdivided into intravascular (within blood vessels) or extravascular (outside of the blood vessels) (Figure 1). Most intrinsic RBC defects are hereditary (for example sickle cell disease, and glucose-6-phosphate dehydrogenase deficiency), whereas most extrinsic causes are acquired (for example antibody mediated-hemolysis and malaria) (Guillaud et al., 2012). Most causes of pathological hemolysis occur in the extravascular compartment, primarily in the spleen. Macrophages and other specialized phagocytic cells of the reticuloendothelial system remove defective RBCs from the circulation. Intravascular hemolysis follows substantial damage to the RBC membrane. An important distinction between these processes is the fate of the RBC contents, particularly the heme moiety of hemoglobin (Hb) and its iron. Iron is an essential nutrient for pathogen and host, and access to iron within the body is the focus of an intense evolutionary battle (Drakesmith and Prentice, 2012; Barber and Elde, 2014). In extravascular hemolysis RBC contents become localized within reticuloendothelial cells, whereas in intravascular hemolysis Hb enters the circulation and can interact with all molecules and cells in contact with the blood (Schaer et al., 2013).

Extracellular Hb causes a variety of adverse clinical outcomes, primarily through NO depletion and free Hb oxidation, which releases free heme (Omodeo-Sale et al., 2010; Baek et al., 2012; Schaer et al., 2013). Accumulation of cell-free heme results in the generation of reactive oxygen species (ROS) and cell damage, eventually causing chronic inflammation, renal dysfunction and vascular disease (Belcher et al., 2010; Gladwin et al., 2012; Schaer et al., 2013). The harmful effects of free heme are abrogated by multiple layers of defense: first the hemoglobin binding protein, haptoglobin; second the heme binding protein, hemopexin; and finally “buffering proteins,” of which the most abundant is albumin (Gozzelino et al., 2010; Schaer et al., 2013, 2014). Haptoglobin binds cell-free Hb, preventing release of its heme moiety, and directing it primarily to monocytes and macrophages expressing CD163 (the haptoglobin receptor) for degradation (Buehler et al., 2009; Schaer and Alayash, 2010). Haptoglobin is upregulated in response to systemic inflammation (Schaer et al., 2014). If levels are depleted by overwhelming hemolysis, then heme may be liberated from free Hb, and heme binds to hemopexin, the next line of defense (Schaer et al., 2014). The hemopexin-heme complex is cleared through receptor-mediated endocytosis, mainly by macrophages and hepatocytes expressing the scavenger receptor CD91 (Hvidberg et al., 2005; Schaer et al., 2014). If the reserve of hemopexin is also overwhelmed, then albumin and lipoproteins bind relatively weakly to cell-free heme, offering an additional buffer against its toxicity (Gozzelino et al., 2010). However, the damaging effects of heme are not only limited by binding, but also by degradation. Heme oxygenase-1(HO-1) is an inducible enzyme that catalyzes the rate-limiting step of heme degradation, converting free heme into iron, carbon monoxide, and biliverdin (Gozzelino et al., 2010). HO-1 expression is induced by its substrate, heme, but also by diverse cytotoxic stimuli, including hypoxia, hyperoxia, ultraviolet radiation, and inflammation (Ryter et al., 2006). The degradation of free heme by HO-1 mitigates the harmful pro-oxidant effects of heme, and the products of heme degradation have important additional cytoprotective effects, one of the most important being limitation of the production of ROS (Bilban et al., 2008; Gozzelino et al., 2010). The many cytoprotective effects of HO-1 are reviewed elsewhere (Ryter et al., 2006; Gozzelino et al., 2010), but one important mechanism is the downstream activation of antioxidant responses, which limit subsequent intracellular production of ROS (Bilban et al., 2008). Expression of HO-1 alters many cellular functions and responses, including responses to inflammatory stimuli and infection (Ryter et al., 2006; Gozzelino et al., 2010). It is debated whether HO-1 is anti-inflammatory *per se* or whether all of its effects can be explained by its cytoprotective actions (Gozzelino et al., 2010).

Whether hemolysis is intravascular or extravascular, much of the Hb-derived heme and iron enters macrophages. The redistribution of the iron from these cells is controlled by hepcidin (Drakesmith and Prentice, 2012), a protein that controls the degradation of the iron efflux transporter, ferroportin (Nemeth et al., 2004; Ganz and Nemeth, 2011). Hepcidin is upregulated in response to infection and inflammation, reducing iron availability for pathogens in blood and tissues, but resulting in sequestration of iron within cells of the reticuloendothelial system, where it may be available to specialized pathogens (Drakesmith and Prentice, 2012).

## Hemolytic infections

The most important infectious causes of significant hemolysis are malaria (Cunnington et al., 2012), Bartonellosis (Minnick et al., 2014), Babesiosis (Gray et al., 2010), and hemolytic uremic syndrome (Kavanagh et al., 2014), and they differ in epidemiology, mechanisms and severity of hemolysis. Other infections may occasionally trigger a secondary autoimmune hemolysis (Guillaud et al., 2012), but will not be considered in detail here.

## Malaria

Malaria, caused by mosquito-transmitted protozoal parasites of the genus *Plasmodium*, is one of the most common infectious diseases in many tropical countries. There were estimated to be 198 million cases in 2013 (World malaria report, 2015), over 80% of these in Africa. The erythrocytic phase of the infection results in repeated cycles of invasion into RBCs, replication within them, and rupture of RBCs to release daughter parasites. A massive increase in both intravascular and extravascular hemolysis occurs, involving both infected and non-infected erythrocytes (Akinosoglou et al., 2012; Safeukui et al., 2015). The amount of hemolysis is related to the parasite load (Cunnington et al., 2012), and is most severe in malaria caused by *Plasmodium falciparum*. The spleen is important for removal of parasitized RBCs from the circulation (Buffet et al., 2009, 2011) and severe anemia (Hb < 5 g/dL) is relatively common in children in highly endemic countries (Cunnington et al., 2013a). Malaria causes the release of Hb into plasma resulting in the formation of Hb-haptoglobin and heme-hemopexin complexes, internalization into monocytes and macrophages, and induction of HO-1 (Pamplona et al., 2007; Ferreira et al., 2008; Yeo et al., 2009; Cunnington et al., 2012). Hepcidin is upregulated in malaria, leading to heme sequestration in macrophages (Drakesmith and Prentice, 2012; Spottiswoode et al., 2014).

## Bartonellosis

Carrion's disease, caused by the sandfly-transmitted intracellular bacterium *Bartonella bacilliformis*, is endemic in the South American Andes (Minnick et al., 2014). It manifests in two remarkably different ways, either as a severe systemic bacteremic illness, Oroya fever, or as a more indolent eruption of blood filled skin lesions (hemangiomas), verruga peruana. Its epidemiology is changing, with a 10-fold increase in cases reported in Peru from 1997 to 2005, and outbreaks occurring in historically non-endemic regions (Minnick et al., 2014). Oroya fever is characterized by intense intra-erythrocytic infection and severe hemolytic anemia (~80% drop in hematocrit). Few studies have investigated the mechanisms of hemolysis in Bartonellosis: increased fragility of red blood cells occurs, but no clear hemolysin has been identified, and it is likely that hemolysis is both intra- and extravascular (Reynafarje and Ramos, 1961; Hendrix, 2000; Minnick et al., 2014).

## Babesiosis

Babesiosis is a relatively uncommon tick-borne infectious disease caused by protozoa of the genus *Babesia* (most often *Babesia microti*). The protozoa invade and replicate within RBCs, producing an appearance which can be very similar to malaria on a blood film (Gray et al., 2010). Most known cases occur in the United States (Centers for Disease Control Prevention, 2012), but Babesiosis is also an emerging infection in Europe, China and other countries (Gray et al., 2010; Hildebrandt et al., 2013; Jiang et al., 2015). The mechanisms underlying the hemolytic anemia are not entirely understood, but there is evidence that both intravascular and extravascular hemolysis occur, with a possible immune-mediated component (Gray et al., 2010). Severe disease is most common in the elderly and immunocompromised (including splenectomised) and whilst anemia is common, severe hemolytic anemia is relatively rare (White et al., 1998; Gray et al., 2010).

## Hemolytic uremic syndrome

Hemolytic uremic syndrome (HUS) is a rare disease, causing substantial intravascular hemolysis and kidney damage, secondary to infection with Shiga toxin-producing *E. coli* and more rarely *Streptococcus pneumoniae* (Kavanagh et al., 2014; Majowicz et al., 2014). Intravascular hemolysis occurs due to thrombotic microangiopathy, where red blood cells become fragmented as they pass through small blood vessels (Kavanagh et al., 2014). Diagnosis of HUS is challenging in resource limited settings.

## Evidence of a causal association with co-infections

Co-infections can only occur when the same population is exposed to both pathogens. Thus, the greatest volume of evidence for an association between infection-related hemolysis and bacterial co-infection comes from studies of *P. falciparum* malaria and NTS (recently reviewed, Takem et al., 2014), which are co-endemic in sub-Saharan Africa. Invasive NTS are amongst the most common bacteria causing invasive disease in children in sub-Saharan Africa, but are rare community-acquired pathogens in Europe and North America (Berkley et al., 2005; Feasey et al., 2012; Ao et al., 2015). Some of the earliest evidence for a causal association between malaria and NTS infection comes from deliberate malaria infections (“malariatherapy”). This was used as a treatment for neurosyphilis before penicillin was available, and an unusually high incidence of invasive NTS infection was observed (Hayasaka, 1933). There is a temporal association of NTS bacteremia with malaria season, even though NTS fecal carriage remains similar throughout the year (Mabey et al., 1987), and a decline in the incidence of NTS has closely mirrored declining malaria transmission in some countries (Mackenzie et al., 2010; Scott et al., 2011). The strongest epidemiological evidence of a causal association comes from a Mendelian randomization study conducted in Kenya, examining the association between malaria, bacteremia, and sickle cell trait (SCT, the asymptomatic carrier state of the sickle cell mutation) (Scott et al., 2011). SCT does not cause hemolysis, and is known to have a strong protective effect against malaria. SCT was found to also be protective against bacteremia with enteric Gram-negative pathogens, conditional on the incidence of malaria. As malaria incidence declined over time, the protective effect of SCT against bacteremia was lost. This study elegantly demonstrated that malaria incidence explained more than half of all bacteremia in this setting. Children with severe malarial anemia or very high parasite load—both indicating the greatest extent of hemolysis—have the highest risk of bacteremia (Bronzan et al., 2007; Hendriksen et al., 2013). Consistent with observations in humans, seminal experimental studies demonstrated increased susceptibility to *Salmonella* infection in mice following hemolysis, either through malaria, chemical or antibody-mediated RBC destruction, but not anemia from blood-letting (Kaye and Hook, 1963a,b; Kaye et al., 1965). Later experiments also demonstrated increased susceptibility to *S. enteritidis, Yersinia enterocolitica*, and *Listeria monocytogenes* associated with hemolysis and specific malaria species (Murphy, 1981; Roux et al., 2010; Cunnington et al., 2011).

A link between Bartonellosis and NTS has also been known for at least 50 years (Cuadra, 1956). In a report of 68 patients with acute hemolytic Bartonellosis in Peru, 35% had an infectious complication, with three of the eight positive blood cultures growing NTS (Maguina et al., 2001). Another observational study recruited 33 patients with acute Bartonellosis, and of the three blood culture positive co-infections *Salmonella* accounted for two, the other being *Klebsiella* (Peregrino et al., 2008). We did not identify any experimental studies of Bartonellosis with bacterial co-infection, so causality remains to be proven, despite suggestive epidemiological evidence.

The relationship between human Babesiosis or hemolytic uremic syndrome and presdisposition to bacterial co-infections does not seem to have been investigated. These conditions are relatively rare and diagnosed most commonly in resource-rich settings where the incidence of community-acquired bacteremia is relatively low. However, Babesiosis is an emerging disease in new geographical regions (Gray et al., 2010; Hildebrandt et al., 2013; Jiang et al., 2015), raising the possibility of co-infections occurring in areas where NTS is more common.

## Mechanisms of hemolysis-induced susceptibility to co-infection

**Figure 2:**
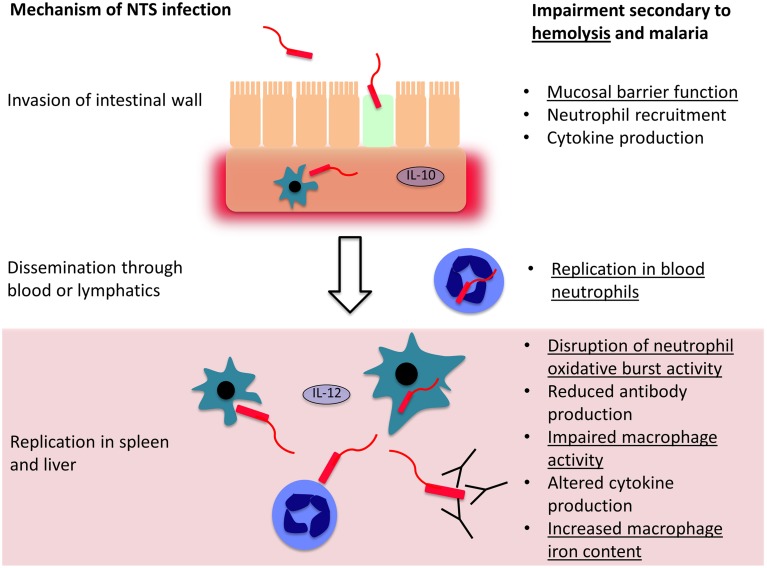
**Mechanisms controlling invasion and dissemination of non-typhoidal** ***Salmonella*** **(NTS)**. NTS invades across the intestinal mucosa, into the submucosal tissues where a local inflammatory cell infiltrate may limit further invasion. If they evade this response, the bacteria disseminate through the blood and lymphatics, and reach phagocytic cells in the spleen and liver, where they may evade killing and replicate. Both hemolysis in general, and malaria, impair host defense mechanisms at each stage of NTS infection. Mechanisms which are likely to be general consequences of intravascular hemolysis are underlined and those that are likely malaria-specific are not underlined.

Most of the understanding of how hemolysis causes susceptibility to bacterial infection comes from *S*. Typhimurium infections in mice, a model which has underpinned most of our understanding of the pathogenesis of invasive *Salmonella* infections (Figure 2). *Salmonella* initially invade epithelial and M cells of the intestine within Peyer's patches (Jones et al., 1994), and bacteria are then phagocytosed by macrophages and dendritic cells in the intestinal wall (Mastroeni et al., 2009; Feasey et al., 2012). This facilitates dissemination via the lymphatic system and blood to other tissues. In the absence of hemolysis, the spleen and liver are the major sites of *Salmonella* replication, where bacteria are predominately found within macrophages (Richter-Dahlfors et al., 1997; Salcedo et al., 2001; Mastroeni et al., 2009). The innate immune response is initially important for control of bacterial numbers, especially phagocytic cell oxidative burst activity (Mastroeni et al., 2000; Vazquez-Torres et al., 2000a). Adaptive immunity, particularly the cell-mediated response, plays a later role in eventual eradication of bacteria (Feasey et al., 2012; McSorley, 2014). In the presence of intravascular hemolysis, bacteria also reach high concentrations in the blood and are found in large numbers, replicating in a new niche within neutrophils (Cunnington et al., 2011).

Relatively few unifying mechanisms have been identified to explain how different causes of hemolysis cause susceptibility to bacterial co-infection (Figure 2). At the level of invasion across the intestinal mucosa, one potential mechanism is hemolysis-related depletion of L-arginine (Chau et al., 2013), the substrate for nitric oxide synthesis. Arginase, an arginine degrading enzyme, is released from RBCs during intravascular hemolysis with free Hb. The latter also drives L-arginine depletion by scavenging nitric oxide (Kato and Gladwin, 2008). L-arginine appears important in limiting intestinal permeability and translocation of both *E. coli* and NTS (Chau et al., 2013). Greater interest has focussed on dysfunction of phagocytic cells—macrophages, and to a lesser extent monocytes and neutrophils. In extravascular hemolysis, erythrophagocytosis has relatively little effect on subsequent phagocytosis of bacteria, but does produce a relatively mild defect in the killing of *Salmonella* by macrophages (Hand and King-Thompson, 1983; Roux et al., 2010). In malarial hemolysis no defect in neutrophil or monocyte phagocytosis has been observed. However, a defect in neutrophil oxidative burst was found, resulting in reduced killing of NTS (Cunnington et al., 2011). This is a particularly compelling explanation for NTS susceptibility, because *Salmonella* have evolved a major virulence mechanism to prevent assembly of the host NADPH oxidase (Vazquez-Torres et al., 2000b), and the oxidative burst is crucial for control of infection (Mastroeni et al., 2000; Vazquez-Torres et al., 2000a). *Salmonella* show a predilection for invasion of neutrophils, presumably, because neutrophil killing mechanisms can be perturbed (Geddes et al., 2007), and accumulation of *Salmonella* in neutrophils is a prominent feature of hemolysis-associated infection (Cunnington et al., 2011). In a mouse model, impairment of the neutrophil oxidative burst was HO-1 dependent (Cunnington et al., 2011). Intravascular hemolysis, due to malaria infection or the chemical hemolytic agent, phenylhydrazine, reduced oxidative burst activity in neutrophils during their development in bone marrow. HO-1 induction occurred in myeloid precursors in bone marrow, and inhibition of HO-1 abrogated the defect in neutrophil oxidative burst and restored resistance to *S*. Typhimurium. This seemingly elaborate mechanism suggests that susceptibility to co-infection is the downside of a carefully orchestrated homeostatic response to limit production of ROS during hemolysis. Free heme plays a central role in the pathogenesis of experimental severe malaria infections in mice, by promoting the production of cytotoxic reactive oxygen species and cell death (Pamplona et al., 2007; Ferreira et al., 2008; Seixas et al., 2009). Presumably ROS produced from neutrophils would exacerbate this toxicity, and reducing production of ROS would be expected to be beneficial. Impairment of the neutrophil oxidative burst, related to upregulation of HO-1, has subsequently been demonstrated in Gambian children with malaria (Cunnington et al., 2012). Impaired neutrophil oxidative burst has also been observed in sickle cell disease (Qari and Zaki, 2011), which is characterized by severe hemolysis and dramatically increased susceptibility to NTS infection (in contrast to SCT), indicating this mechanism may be a general consequence of hemolysis. Several important questions remain unanswered: which heme containing moiety (and via which receptors) leads to HO-1 induction during neutrophil development? At which stage in neutrophil development does this HO-1 induction produce its effect? And, how exactly does HO-1 induction lead to suppression of the oxidative burst?

Other mechanisms of susceptibility to co-infection have been extensively investigated in malaria (Takem et al., 2014). Proposed mechanisms include impairment of: microvascular blood flow and gut mucosal barrier function; innate and adaptive intestinal immunity; antibody production; and splenic, macrophage and neutrophil function. In *P. falciparum* malaria, parasitized red blood cells stick in small blood vessels (Cunnington et al., 2013b) in many tissues including the gut, resulting in obstruction, localized tissue hypoxia and ischemia (White et al., 2013), which could impair mucosal barrier function (Berkley et al., 2009). Malaria infection in mice causes IL-10 dependent attenuation of neutrophil migration into the intestinal mucosa in response to *Salmonella* infection, removing another line of defense (Lokken et al., 2014; Mooney et al., 2014). Macrophage dysfunction occurs secondary to ingestion of hemozoin, the insoluble hemin polymer generated by parasites during hemoglobin digestion (Boura et al., 2013), inhibiting oxidative burst activity, expression of MHC-class-II, phagocytosis and killing of bacteria (Schwarzer et al., 1992, 1998; Schwarzer and Arese, 1996), and IL-12 secretion (Keller et al., 2006). IL-12 is important in control of Th1 responses and reduced expression increases susceptibility to *Salmonella* (MacLennan et al., 2004). Malaria suppresses heterologous antibody responses (Cunnington and Riley, 2010), and antibodies are an important component of defense against NTS (MacLennan et al., 2008; Gondwe et al., 2010). Alterations in spleen function and accumulation of iron in macrophages may also be important (van Santen et al., 2013; Gomez-Perez et al., 2014). To our knowledge mechanisms for the increased susceptibility to co-infection in Bartonellosis have not been investigated. Of the proposed mechanisms above, we believe that HO-1 dependent neutrophil dysfunction provides the most plausible explanation for susceptibility to co-infection occurring during hemolysis in malaria, Oroya fever and non-infectious causes in experimental models.

## Prevention of co-infection and co-morbidity

Malaria and Bartonellosis can be diagnosed rapidly where appropriate resources exist (Minnick et al., 2014; World malaria report, 2015) but diagnosis of bacterial co-infection is more difficult, both clinically (because the pre-existing infection may mask signs and symptoms of secondary infection) and in the laboratory (because blood cultures are required, with a 24–48 h incubation period) (Takem et al., 2014). Identification of markers to stratify risk of co-infection could allow those at highest risk to receive empirical antibiotic prophylaxis or treatment. Quantitative measurement of *P. falciparum* histidine rich protein 2 (PfHRP2), is a promising marker to identify those with malaria at risk of co-infection (Hendriksen et al., 2013). Another strategy would be to modulate HO-1 activity, since tin protoporphyrin can reverse HO-1–mediated neutrophil dysfunction (Cunnington et al., 2011), but only after treatment of the hemolytic infection in order to avoid exacerbated heme toxicity. The greatest reduction in co-morbidity will undoubtedly come from public health measures to control or eliminate the hemolytic infections. Malaria control is already known to produce dramatic decreases in the population burden of NTS bacteremia (Mackenzie et al., 2010; Scott et al., 2011) and all-cause child mortality (Kleinschmidt et al., 2009). Control of Carrion's disease appears more challenging, and indications of expanding geographical distribution suggest that co-infections may remain a problem for the foreseeable future (Minnick et al., 2014).

## Conclusion

A causal link between infection-related hemolysis and bacterial co-infection has been established for the association of malaria and NTS. Further research is needed to confirm whether the same mechanism applies in Oroya fever, and whether Babesiosis will predispose to co-infection in the same way. This may also give us greater insight into bacterial infections occurring in non-infectious hemolytic diseases, such as sickle cell disease. Although infection prevention is likely to have the biggest impact on mortality, a greater mechanistic understanding may allow targeted interventions to those most at risk of bacterial co-infection.

### Conflict of interest statement

The authors declare that the research was conducted in the absence of any commercial or financial relationships that could be construed as a potential conflict of interest.
